# Temporal coherency of mechanical stimuli modulates tactile form perception

**DOI:** 10.1038/s41598-021-90661-1

**Published:** 2021-06-03

**Authors:** Masashi Nakatani, Yasuaki Kobayashi, Kota Ohno, Masaaki Uesaka, Sayako Mogami, Zixia Zhao, Takamichi Sushida, Hiroyuki Kitahata, Masaharu Nagayama

**Affiliations:** 1 Faculty of Environment and Information Studies, Keio University, Tokyo, Japan; 2 Research Institute for Electronic Science, Hokkaido University, Sapporo, Japan; 3 Graduate School of Mathematical Sciences, The University of Tokyo, Tokyo, Japan; 4 Faculty of Policy and Management, Keio University, Tokyo, Japan; 5 Department of Computer Science and Technology, Salesian Polytechnic, Machida, Japan; 6 Graduate School of Science, Chiba University, Chiba, Japan

**Keywords:** Membrane biophysics, Applied mathematics, Sensory systems, Somatic system, Human behaviour

## Abstract

The human hand can detect both form and texture information of a contact surface. The detection of skin displacement (sustained stimulus) and changes in skin displacement (transient stimulus) are thought to be mediated in different tactile channels; however, tactile form perception may use both types of information. Here, we studied whether both the temporal frequency and the temporal coherency information of tactile stimuli encoded in sensory neurons could be used to recognize the form of contact surfaces. We used the fishbone tactile illusion (FTI), a known tactile phenomenon, as a probe for tactile form perception in humans. This illusion typically occurs with a surface geometry that has a smooth bar and coarse textures in its adjacent areas. When stroking the central bar back and forth with a fingertip, a human observer perceives a hollow surface geometry even though the bar is physically flat. We used a passive high-density pin matrix to extract only the vertical information of the contact surface, suppressing tangential displacement from surface rubbing. Participants in the psychological experiment reported indented surface geometry by tracing over the FTI textures with pin matrices of the different spatial densities (1.0 and 2.0 mm pin intervals). Human participants reported that the relative magnitude of perceived surface indentation steeply decreased when pins in the adjacent areas vibrated in synchrony. To address possible mechanisms for tactile form perception in the FTI, we developed a computational model of sensory neurons to estimate temporal patterns of action potentials from tactile receptive fields. Our computational data suggest that (1) the temporal asynchrony of sensory neuron responses is correlated with the relative magnitude of perceived surface indentation and (2) the spatiotemporal change of displacements in tactile stimuli are correlated with the asynchrony of simulated sensory neuron responses for the fishbone surface patterns. Based on these results, we propose that both the frequency and the asynchrony of temporal activity in sensory neurons could produce tactile form perception.

## Introduction

The human hand can detect both form (geometric) and texture information of a contact surface^1^. For example, surface geometry edges are detected when a tactile stimulus is presented on a finger pad. Human observers tend to actively scan the contact surface when they examine the surface texture roughness^2,3^. These results illustrate that human perceivers can use skin displacement and changes in skin displacement to detect and recognize the world outside the body through touch modality.

A previous study showed that tactile form perception and coarse roughness perception are mediated by slowly adapting type I afferents, which detect skin displacement^4–6^. Recent literature shows that fine roughness perception is mediated by rapidly adapting afferents (RA and PC afferents), which detect temporal changes in skin displacement (e.g., vibration)^6^. The detection of skin displacement and changes in skin displacement are thought to be conveyed in different tactile channels; however, tactile form perception may use both types of information. For example, force direction and geometric cues are encoded in the first spike of RA and SA-I afferents^7,8^.

**Figure 1:**
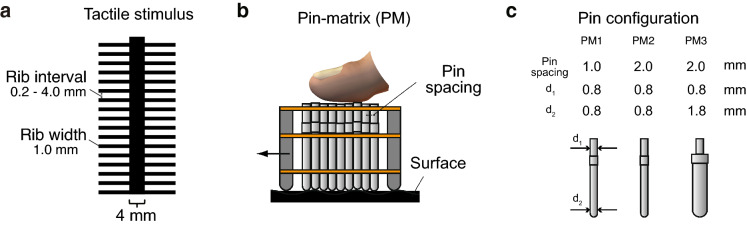
Geometric profile of tactile stimuli used in psychological experiments. (**a**) Geometric configuration of the fishbone tactile illusion (FTI) pattern. This geometry comprises a central strip (the spine of the fishbone) and ribs (adjacent areas). In the experiment, the central strip width was constant, but the interval between ribs was varied to change the perceived stimulus temporal frequency in the adjacent rib areas. (**b**) Schematic of the passive pin matrix. This instrument comprises pins of 0.8 or 1.8 mm in diameter, with a pin spacing of 1.0 or 2.0 mm. The pins can follow the surface geometry beneath the pin matrix in the vertical direction and suppress the horizontal displacement. (**c**) We prepared three pin matrices with multiple pin configurations; $d_1$ indicates the diameter of the contact area to the fingertip, and $d_2$ indicates the diameter of the contact area to the surface geometry.

As in vision research, the study of perceptual phenomenon can also provide valuable insights on how tactile information processing is achieved^9–11^. In touch, the fishbone tactile illusion (FTI) is a perceptual phenomenon in which a person feels an indented geometry even though the surface being felt is almost flat geometrically^12^ (Fig. 1a). This phenomenon occurs because the flat smooth surface is surrounded by rough texture. Previous research has shown that this phenomenon occurs when relative motion between the contact surface and fingertip exists, suggesting that not only skin displacement but also changes in skin displacement can produce tactile form perception^13,14^.

The FTI has been discussed as a variant of the ridge/trough illusion^9,10^. Hayward noted that juxtaposing surfaces that have different mechanical properties such as adhesion or friction can produce the ridge/trough illusion. In this illusion, the tactile system tends to interpret variations in surface frictional and adhesional properties as variations in surface geometry^10^. Moreover, when the lateral regions of fingertip skin is strained by traction loading in a quasistatic manner while the central region is stationary, the concave trough illusion occurs. The principle of this tactile illusion in a quasistatic condition is discussed based on the the phenomenological similarity of the spatial strain distribution in the deeper layer of the finger tissue^15,16^.

Interestingly, Oyarzábal et al. showed another variant of the ridge/trough illusion^13^. This variant is produced with using a tactile shape display composed of a $6 \times 6$ array of pins^17^. Participants could not perceive a 0.1 mm deep central indentation when it was presented statically, but it was readily detected when the pattern was vibrated at 5 Hz with an amplitude of 0.1 mm. In comparison with the typical variants of the ridge/trough illusions, Oyarzábal’s variant provides a periodically changing tactile stimulus of 0.1 mm vertical displacement. This example shows a possibility that the ridge/trough illusion, or at least the FTI, can be produced by both quasistatic, spatial deformation and dynamic, spatiotemporal deformation of skin tissue.

Spatiotemporal deformation of skin tissue is thought to be used for motion detection^18,19^. In vision, motion information is used to encode the third dimension, that is, the perception of depth^20^. Because of the similarity of motion processing in vision and touch^21^, one may expect that there would also be similar functional benefits of motion processing in depth perception in touch. Briefly, the source of motion information, which is the spatiotemporal deformation of skin tissue, can be used for the detection and the assessment of depth in touch.

Therefore, we hypothesize that there could be another mechanism that can produce the ridge/trough illusion. One mechanism is based on the spatial cue encoded in SA I afferents as proposed in previous literature^16^, and another mechanism is the spatiotemporal cues encoded in RA afferents. Both mechanisms may contribute to the occurrence of the ridge/trough illusion. We predict that the magnitude of the perceived surface indentation reduces as the temporal asynchrony of tactile stimuli decreases. In other words, temporal coherency of tactile stimuli can change the tactile form perception due to the absence of motion cues.

To explore the contribution of the spatiotemporal change in skin displacement to tactile form perception, we first conducted a psychological study to test whether the attenuation of horizontal displacement affects tactile form perception using an apparatus that can extract only the vertical displacement of the contact surface at specific spatial resolutions to mimic the experimental stimulus presented by a tactile shaped display of pin arrays^13^. The contactors of this apparatus are similar to those of actuator-driven pin arrays^22,23^ but in the form of passive mechanical components so that we do not need to consider the temporal resolution of the presented tactile stimulus. We found that human participants experienced the attenuation of perceived surface indentation in the FTI in a certain condition, where tactile stimulus was spatially coherent. Second, to explain the attenuation of the tactile illusion, we developed a sensory neuron model by combining the ion conductance model of mechanosensitive channels^24^ and the Hodgkin–Huxley model^25^ to simulate the response of the receptive field that detects the change in skin displacement. Using the developed mathematical model, we tested whether the spatially coherent tactile stimulus could produce synchronized responses in tactile receptive fields. By combining the results of psychological experiments and computational analysis, we propose that human form perception in fingertips is produced by both skin displacement (spatial) information and the change in skin displacement (spatiotemporal) information encoded in tactile sensory neurons.

## Results

### Psychological experiment

Human participants feel the strongest magnitude from the FTI under certain conditions upon examination by a bare finger. We prepared nine different fishbone surfaces by changing the rib intervals in adjacent areas (Fig. 1a). The participants compared pairs of stimuli randomly chosen from a set of $9 \times 4 = 36$ elements representing all combinations of rib interval under the same touch condition. The participants did not compare pairs of stimuli under different touch conditions (i.e., bare finger vs pin matrix 1, pin matrix 1 vs pin matrix 2, etc). We asked participants to answer which surface felt more indented when they were presented a pair of fishbone surfaces of different rib intervals in a two-alternative forced choice fashion.

**Figure 2:**
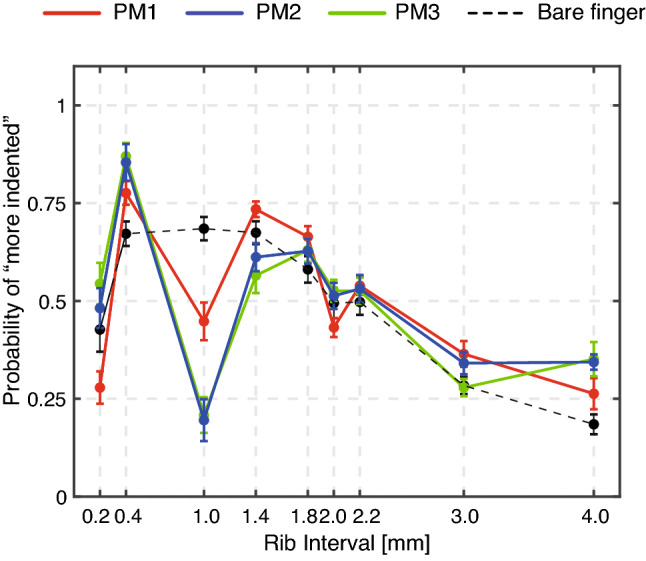
The touch condition affects the mean probability of perceiving a more indented (hollower) surface geometry. Participants examined a pair of different fishbone patterns (36 combinations in total), and they indicated which pattern was more indented in each trial under a certain touch condition (bare finger or utilizing pin matrix (PM) 1, 2, or 3. Psychological data showed different probabilities of answering “more indented” than other rib intervals ($N=12$). The horizontal axis represents the rib interval in the fishbone surface pattern. Under bare-finger conditions, the probability increased between 0.2 and 0.4 mm and gradually decreased when the rib interval exceeded 1.4 mm. The probability trace was exaggerated under pin matrix conditions. Specifically, the probability steeply increased between 0.2 and 0.4 mm and decreased as the rib interval increased under touch conditions with PM1, except for the steep dips at rib intervals of 1.0 and 2.0 mm. This trend was more enhanced under touch conditions with PM2 and PM3. In particular, the probability of more indented was abruptly decreased at the 1.0 mm rib interval. Mean estimates of probability are plotted, and error bars indicate $95 \%$ confidence intervals.

Psychological data (*N*=12, Fig. 2) shows the probability that a certain surface form felt more indented by comparing a pair of fishbone patterns of different rib intervals. The horizontal axis indicates the rib interval, and the vertical axis indicates the probability of choosing “more indented” for that rib interval. Under bare-finger conditions, the probability maintained maximum values at rib intervals of 0.4–1.4 mm (Fig. 2). The probability decreased with increasing rib interval over 1.8 mm.

The same data were also analyzed by using the method of pairwise comparisons. Supplementary Fig. S1 was plotted by polling all of the data from twelve participants who conducted the comparison task for each pair. The participants felt the strongest magnitude of the perceived surface indentation in certain conditions upon examination by a bare finger. The probability of answering “more indented” is equivalent to the relative magnitude of indentation, so in the following, we use the relative magnitude of perceived surface indentation to describe the strength of indented form perception.

The relative magnitude of indentation in the FTI can be modulated by using a pin matrix. This apparatus has multiple straight pins that move passively up and down according to the height of the geometric surface beneath the pin matrix (Fig. 1**b**)^12^. The advantage of using a pin matrix is that the experimenter can change the spatial frequency of the tactile stimulus arbitrarily by changing the interval between adjacent pins (Fig. 1c). Moreover, we could attenuate the amplitude of horizontal displacement of tactile stimuli due to the mechanical constraint of passive pins. We prepared two different kinds of spacing between pins (1.0 and 2.0 mm) and two different pin diameters (0.8 and 1.8 mm) and conducted the same experiment as with the bare-finger condition (see Fig. 1c for configurations). By using a pin matrix with a spacing of 1.0 mm (PM1), the perceived relative depth of the perceived surface indentation was exaggerated.

Pin matrix (PM) 2 and PM3 had a center-to-center spacing of 2.0 mm. Under both PM2 and PM3 touch conditions, a spatial aliasing of tactile stimuli occurred when the rib interval of the fishbone pattern was 1.0 mm. In other words, all the pins above the fishbone rib were displaced synchronously due to the spatial aliasing. We prepared two kinds of pins whose diameters of the area contacting the surface geometry were 0.8 mm (PM2) and 1.8 mm (PM3) to test whether the relative amount of pin displacement would affect the relative magnitude of the perceived surface indentation. Psychological test results showed that the relative magnitude of the perceived surface indentation was attenuated when all the pins moved synchronously (under PM2 and PM3 touch conditions). We did not observe an apparent difference in the overall trend between PM2 and PM3 touch conditions. Strikingly, the effect of modulation produced by pin matrices was even larger when a perceiver used a pin matrix with a spacing of 2.0 mm (PM2 and PM3).

We observed two phenomena under the utilization of the pin matrices. First, the ridge/trough illusion occurred under the pin matrix conditions (Fig. 2; see also Table 1). Second, the probability of describing the 1.0 mm rib interval as “more indented” decreased when the participants examined the surface with pin matrices of a 2.0 mm spatial interval (PM2 and PM3) compared with a 1.0 mm spatial interval (PM1).

The psychological experiment raised two important questions for tactile form perception in humans. (1) Does the temporal frequency of sensory neuron responses produced by fishbone rib influence and/or (2) does the temporal asynchrony between sensory neurons affect the relative magnitude of perceived surface indentation in the FTI?

For the first question, we could predict that the narrower the rib interval becomes, the larger the responses of sensory neurons become. Therefore, we could assume that the temporal frequency of sensory neuron responses increases by decreasing the interval of pin spacing in the pin matrices. Does the frequency of action potentials account for the relative magnitude of the perceived surface indentation?

For the second question, when using the 2.0-mm pin matrices (PM2 and PM3), all the pins in the rib areas moved up and down simultaneously when a perceiver touched the surface with rib intervals of integers (1.0, 2.0, 3.0 and 4.0 mm). In this mechanical configuration of tactile stimuli, coherent information of tactile signals could be encoded in the responses of sensory neurons. The simplest idea is that the coherent tactile signal would be encoded as the synchronous response of sensory neurons. If we could observe the correspondence between the tactile stimuli and the neural responses in terms of synchrony, we would be able to account for the psychological result in terms of neural responses. Consequently, we could assume that the temporal asynchrony of the responses in tactile sensory neurons corresponds to the magnitude of perceived surface indentation in the FTI.

To address these questions, we developed a computational model of a tactile sensory neuron based on previous literature. With this model, we calculated both the temporal frequency and the asynchrony of responding tactile sensory neurons, and we conducted a correlation analysis with the psychological data.

### Computational experiment

**Figure 3:**
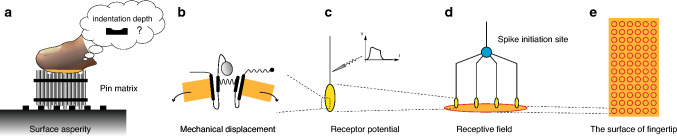
Mathematical model. (**a**) A pin of the pin matrix produces skin deformation that leads to (**b**) opening a mechanosensitive ion channel in a mechanoreceptor. (**c**) Change in receptor potential due to the mechanoreceptor’s physiological response is conveyed electrically to a spike initiation site (**d**) where four mechanoreceptors are connected. This mechanoreceptor-sensory neuron system works as (**e**) a responsive receptive field distributed uniformly in the fingertip. After calculating the responses of multiple receptive fields, both the firing rate of each receptive field and the firing timing synchronicity (the Shannon entropy) over multiple receptive fields are calculated. The concept of the process of mechanoreception is developed based on proposals in the existing literature^29,38,39^.

We developed a simple computational model that captures sensory neuron responses in the peripheral nervous system. Figure 3 shows a schematic of our computational model of how the receptive fields respond to mechanical input. This mathematical model has a hierarchical structure that is composed of multiple stages of touch reception. In short, the mechanical deformation produced by touching the pins with a fingertip leads to the activation of the mechanosensitive channels of mechanoreceptor (Fig. 3a, b). This assumption is based on the reports that physical quantities related to local membrane stretch can predict sensory neuron responses^26^. The responses of multiple mechanoreceptors are integrated into one sensory neuron response at a spike initiation site (Fig. 3c, d). In the simulation, we assumed that the same type of receptive-field mechanoreceptor system is distributed uniformly in the fingertip (Fig. 3e; see “Materials and methods” for details). We consider that modeling the sensory neuron responses from the behaviors of mechanosensitive ion channels is critical because we assume that the correspondence between the timing of mechanical input and the timing of sensory neuron responses is required for elucidating the attenuated depth form perception under pin-matrix conditions (Fig. 2).

**Figure 4:**
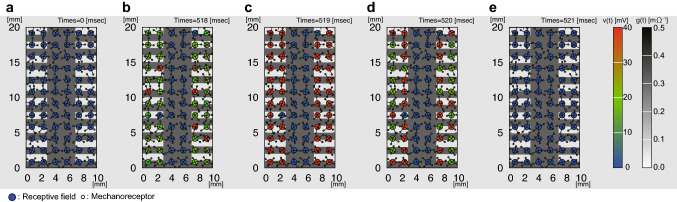
Numerical results of receptive fields at different time points. (**a**) Initial setup. (**b–e**) Four successive time moments [$t=$518 (**b**), 519 (**c**), 520 (**d**), and 521 ms (**e**)], where typical responses of mechanoreceptors to mechanical input signals are shown. Large circles represent receptive fields, whose action potential activities *v*(*t*) are indicated by the color bar; red and blue represent the excited and the resting state, respectively. Each receptive field has a neuron that integrates signals from mechanoreceptors, which are represented by small circles. The activities of the mechanoreceptors and the conductance *g*(*t*) are represented by the grayscale color. Connections between a receptive field and mechanoreceptors are indicated by lines. After calculating the responses of multiple receptive fields, both the firing rate of each receptive field and the firing timing synchronicity (the Shannon entropy) over multiple receptive fields are calculated. Supplementary Videos 1–3 demonstrate the time-lapse results of how 72 receptive fields responded to the fishbone pattern of 1.0 mm and 0.4 mm intervals under PM1 and PM2 conditions.

We performed numerical experiments corresponding to the PM1 condition with two different rib intervals. A representative result of the numerical simulation is shown in Fig. 4 and Supplementary Fig. S4. Spatial event plots revealed that the occurrence timing of action potentials was more synchronized for the 1.0 mm rib interval than for the 1.4 mm rib interval. This qualitative result may explain the decrease in the relative magnitude of perceived surface indentation observed in the psychological experiment (Fig. 2).

### Correlation analysis between psychological data and computational result

To explain our psychological results, we conducted a correlation analysis to evaluate the effect of temporal frequency and temporal asynchrony of sensory neuron responses on the psychological data. To evaluate the effect of the temporal frequency of sensory neuron responses, we adopted the averaged number of responses from sensory neurons in adjacent areas (fish ribs) to gauge the contribution to the sensory evaluation results. To evaluate the effect of temporal asynchrony, we employed the Shannon entropy of the probability of action potential occurrence. If the Shannon entropy has a small value, the temporal asynchrony of action potential occurrence is low, implying that multiple tactile receptive fields respond simultaneously.

**Figure 5:**
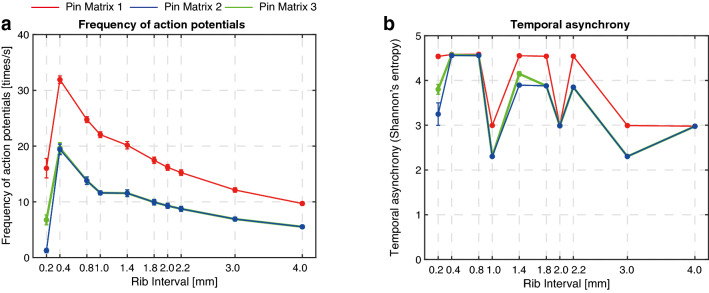
Detailed analysis of numerical results. (**a**) In our model, the sensory neuron responds vigorously to the fishbone patterns when the rib interval is 0.4 mm for all pin matrix conditions. (**b**) Calculated temporal asynchrony of action potentials. There are sudden dips in the calculated values at rib intervals of 1.0, 2.0 and 3.0 mm.

Figure 5 shows numerical results when tactile stimuli were presented through PM1 (1.0-mm pin spacing), PM2 and PM3 (2.0-mm pin spacing) for different rib intervals. Figure 5a shows the averaged frequency of action potentials of all 72 receptive fields. The simulation data indicate that the maximum frequency of action potentials from sensory neurons is obtained at a rib interval of 0.4 mm, which coincides with the rib interval at which the maximum relative magnitude of perceived surface indentation is observed (Fig. 2). The same data, however, do not correspond to the sudden dips of the indentation magnitude at 1.0 mm and 2.0 mm rib-interval conditions. On the other hand, the temporal asynchrony of sensory neuron responses as characterized by the Shannon entropy decreased under the 1.0 mm and 2.0 mm rib-interval conditions (Fig. 5b). This computational result implies that the responses of sensory neurons were synchronized when significant dips of relative magnitude of perceived surface indentation were observed.

The correlation analysis revealed that the Shannon entropy corresponds with the psychological result. Kendall and Spearman’s rank coefficients were significantly correlated between the Shannon entropy ranking and the perceived indentation ranking for all pin matrix conditions (Table 2). However, these rank correlation coefficients were significantly correlated between the frequency of action potential event ranking and perceived indentation ranking only for the PM1 condition. This correspondence between the perceptual phenomenon and the computational model (frequency and asynchrony of sensory neuron responses) may explain the lower probability of perceived surface indentation at 1.0 mm and 2.0 mm rib intervals, as shown in Fig. 2. In particular, the lower the Shannon entropy values are, the lower the relative magnitude of perceived surface indentation.

## Discussion

We reported that the relative magnitude of depth perception in the fishbone pattern decreased at specific ridge intervals under pin matrix conditions (Fig. 2). Attenuation of relative magnitude was not found in the bare-finger condition, suggesting that a group of vertical displacements of tactile stimuli that were spatially discrete could produce this phenomenon.

The ridge/trough illusion has several variants. This illusion can be produced by adhesional or frictional surfaces^9,10,14^. Hayward discussed the process by which mechanical boundary conditions are translated into perceptual boundary conditions by noting that the tactile system is capable of reporting the simplified representation of mechanical deformation in actual finger tissue. This explanation was studied by a finite element analysis of deformations in finger tissue that are produced by normal loading and tangential loading, and similar strain distributions were observed in the layer of skin tissue where mechanoreceptors are located^16^. It is expected that other variants of the ridge/trough illusion will ultimately be explored to understand the mechanisms underlying tactile form perception.

In the present study, we evaluate the pin array version of the FTI as a variant of the ridge/trough illusion. This is an intriguing variant because this pin-array version provides spatiotemporal deformation of skin tissue. Such skin deformation can produce the perception of motion. Interestingly, a previous neurophysiological study reported that RA (FA I) and PC (FA II) afferents were activated by the pin-array tactile display, the modified version of OPTACON, but did not report any responses from SA I afferents^27^. Even though we cannot directly infer a conclusion without physiological recording from sensory afferents, the pin-array version of the FTI can be produced by the responses from RA afferents. RA afferents are thought to be responsible for the asynchronous information of spatiotemporal low-frequency vibrations in the finger pad based on psychophysics results^18^. We considered it important for elucidating our psychological data that the asynchrony of RA afferent responses are generated by the tactile stimulus.

To explain what we observed in the psychological experiments, we developed a simple computational model of RA sensory neuron responses by integrating the ion conductance model of mechanosensitive channels in the cell membrane^24^ and the Hodgkin–Huxley model^25^. We assumed that four mechanoreceptors were connected to a spike initiation zone where the sum of ion currents was integrated (Supplementary Fig. S2) based on previous literature on anatomical structures of peripheral nerves in finger pads^28^.

A recent study on computational models of slowly adapting touch receptors included a hypothesis that the generator current coming from mechanoreceptors integrates at a spike initiation zone^29,30^. Lesniak and Marshall et al. proposed that neural spikes are generated at a spike initiation zone, where the generator currents from mechanoreceptors are integrated. In a follow-up study by the same group, Gerling et al. formulated a function for a generator current by combining slowly, rapidly, and ultraslowly inactivating currents from a mechanoreceptor unit^30^. Their computational model used a leaky integrate-and-fire model to simulate neural dynamics. We adopted the Hodgkin–Huxley model in the computational analysis to generate action potentials at a spike initiation zone in each receptive field without assuming the spike initiation threshold. Another RA model uses probability theory to reconstruct the firing rate of the sensory neuron, but it does not consider the exact temporal timing of RA responses^31^. We consider it important to model both firing rates and temporal timing of RA responses in this study.

We arranged 72 low-threshold mechanoreceptive fields uniformly to calculate the ensemble of sensory neuron responses according to the mechanical displacements of the surface pattern presented through the pin matrices (Fig. 3, Supplementary Figs. S4, S5). Note that the locations of mechanoreceptors were randomized so that the responses of the receptive fields could capture the intrinsic property of mechanoreception. The mechanoreceptive fields were uniformly arranged for the ease of visual presentation of group responses of sensory neurons. We calculated the temporal frequency and asynchrony of computed sensory neuron responses (Fig. 5). We found that temporal asynchrony of sensory neurons could explain our psychological experiment (Table 2).

Even though our mathematical model does not fully depend on neurophysiological data, as in a previous study^32^, our computational model could capture the human nature of tactile form perception, at least of the FTI.

### Origin of temporal asynchrony in computed sensory afferent responses

**Figure 6:**
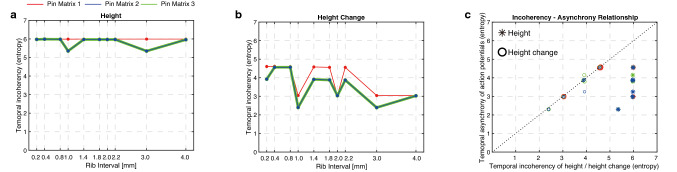
Calculated incoherency of mechanical displacements through pin matrices. The temporal incoherency of (**a**) height and (**b**) height change was indexed by the Shannon entropy. (**c**) Relationship between incoherency of tactile stimuli through pin matrices and temporal asynchrony of action potentials for different pin matrix conditions.

The temporal asynchrony of computed sensory afferent responses originates from the temporal incoherence of tactile stimuli. We calculated the timing of maximum displacement of tactile stimuli presented by different pin matrices (Supplementary Fig. S5) and calculated the temporal incoherences of tactile stimuli using the same metric (Shannon entropy, equations 13) as for the temporal asynchrony of computed sensory afferent responses. We computed the temporal incoherence of the displacement and displacement changes of pins in pin matrices (Fig. 6a, b). We found that the temporal incoherence of displacement changes showed similar trends as the temporal asynchrony of computed sensory afferent responses (Fig. 6c). This result implies that the temporal incoherences of displacement changes presented on to the skin may explain the relative magnitude of perceived surface indentation in the FTI.

**Figure 7:**
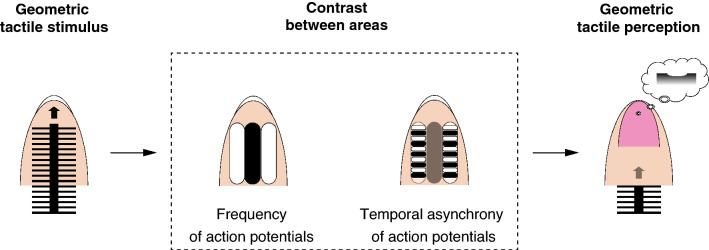
A model of tactile form perception in fingertips. Both the frequency and the temporal asynchrony of action potentials may contribute to tactile form perception. If the temporal asynchrony of action potentials were to decrease, a perceiver would report attenuated depth of perceived surface indentation at the backbone of fishbone patterns.

A previous study reported that human participants showed a lower detection rate for incoherent tactile stimuli after exposure to vibrotactile stimuli^18^. This previous study implies the existence of a temporal asynchrony (and a motion) detector in humans. Kuroki and Nishida^18^ assumed that the detection of asynchrony of tactile stimuli is used in perceiving a moving tactile stimulus, and we agree with their idea. In addition, our data imply that the detection of asynchrony of tactile stimuli is also used in tactile form perception. Based on our psychological result, we propose a model for tactile form perception in humans (Fig. 7). The rank of perceived magnitude of indentation was correlated with the rank of temporal frequency of simulated neuron under the PM1 touch condition but was not correlated under PM2 and PM3 conditions. On the other hand, the rank of relative magnitude of perceived surface indentation was correlated with the rank of the Shannon entropy under all of the PM conditions with statistical significance (Table 2). Our working model proposes a hypothesis that both temporal frequency and temporal asynchrony of action potentials may play a role in tactile form perception.

### The computational model predicts that a tactile sensory afferent system that can detect changes in displacement may play a role in tactile form perception

The correlation analysis showed that the responses of tactile sensory channels (i.e., RA afferents) that detect changes in displacements can mediate information concerning tactile form perception. In general, all four kinds of tactile sensory afferents in the glabrous skin of the human hand respond to the onset of mechanical input, which is equivalent to the change in displacement^33^. For scanned surface patterns, Blake et al. recorded the responses of SA I and RA mechanoreceptive afferents innervating the glabrous skin of the rhesus monkey and reported that both sensory afferents responded at the edges of scanned raised surfaces^34^. However, Gardner and Palmer reported that only RA and PC mechanoreceptors could respond to the vibrotactile stimuli presented by the modified version of OPTACON, whose maximum displacement was approximately 0.065 mm^27^. The tactile stimulus presented in this study was less than 0.02 mm of vertical displacement, so we assume that the RA sensory afferents play a role in tactile form perception, at least under the pin-array variant of the ridge/trough illusion.

A recent study of surface feature detection in first-order tactile neurons proposed the involvement of the temporal structure of a sensory afferent neuron. Johansson and Birznieks reported that the first spikes of human tactile afferents can encode the direction of applied force^8^. Pruszynski and Johansson reported that both the temporal frequency (intensity) and the temporal structure of two types of first-order tactile neurons (SA I and RA) could explain the edge orientations of tactile forms scanned across a human fingertip^35^. Similarly, neurophysiological data and computational data of modeled sensory neuron responses support the view that millisecond-precision spike timing is utilized to perceive skin vibration^32,36^.

Based on our results, we assume that RA can affect tactile form perception as well as SA I. Although a group of studies supported the hypothesis that tactile form perception is mediated by SA I^1^, surface texture is also encoded in both SA I and RA^4,5^. Weber et al.^6^ also reported that RA could encode both spatial and temporal variations in natural textures. These results support our view.

Although we cannot address the question of whether SA I and/or RA mediate tactile form perception is produced by the FTI based on the current computational model, we suggest that RA channels can encode the temporal incoherence of tactile stimuli. Additionally, the density of receptive fields simulated in our computational model is similar to the neurophysiological arrangement of receptive fields in RA afferents^28^. This view is worth addressing by conducting neurophysiological recordings^33^ or simulating with established computational models^32^ by presenting tactile stimuli equivalent to both SA I and RA channels in a future study.

### Relationship between the coherence of stimulus and form reconstruction in vision and touch

Synchronous displacements of tactile stimuli attenuated the relative magnitude of the perceived surface indentation. Synchronous displacements occurred because of the spatial aliasing under PM2 and PM3 conditions. Since these pin matrices are passive mechanical filters, the temporal resolution of tactile stimuli is continuous. Computationally, it is possible to reconstruct physical surface geometry by integrating spatial and temporal information of pin displacements. Therefore, the attenuation of the FTI under PM2 and PM3 conditions cannot be ascribed to the spatial aliasing alone. Conversely, this psychological data suggested that critical information in tactile signal processing of form perception was lost due to the synchronous displacements.

Incoherent tactile stimulus produces the perception of asynchrony, which is a source of motion detection^18^. In other words, synchronous displacements of tactile stimuli lead to the attenuation of motion perception. In vision, a spatiotemporally incoherent stimulus produces motion perception, and motion cues are used to reconstruct the 3D structure of scenes (i.e., depth) in the real world^20^. The similarity of sensory signal processing between touch and vision has previously been discussed^21^; we assume the motion perception in touch can be used to reconstruct the 3D structure of tactile stimuli, i.e., the contacting surface geometry. To our knowledge, there has been no research on the effect of motion cues in tactile form perception, and this is a topic of planned research.

### Limitations of this study

In our model, we considered only the displacement of pins as mechanical input but did not take elastic deformation of the skin into consideration. Mechanoreceptors that form tactile receptive fields are embedded in the skin, which consists of multiple layers of different mechanical properties. If we could take the elastic effect of the skin into account in our model, we might be able to establish a mathematical model that realistically reconstitutes the observed neural responses to indented^26,37^ and/or scanned forms^34^ observed in previous neurophysiological recordings. We consider that our computational model is applicable to explain, at least, the decreased magnitude of perceived surface indentation in the FTI through pin matrices.

## Conclusion

In summary, this study shows that spatial contrast alone cannot fully explain the form perception in the pin-array version of the FTI, which is a variant of the ridge/trough illusion. Temporal synchronicity of the tactile stimuli corresponds to the rank of perceived surface indentation. We predict that sensory neuron responses (indexed by the Shannon entropy) may explain the diminished phenomenon in addition to spatial-rate coding theory. Our mathematical model remains valid for explaining the tactile form perception, at least in the range of small mechanical deformation up to 20 $\upmu$m used in the current study. The next study will address whether we can control the relative magnitude of perceived surface indentation by changing the temporal incoherence of tactile stimuli and see the correspondence between psychological result (perceived magnitude of geometric indentation) and temporal incoherence as an index of tactile form cues.

**Table 1:** Statistical analysis of the effect of rib intervals in each touch conditions (bare finger, PM1, 2, and 3) (see Fig. 1a).

	*F* value	*p* value	$\upeta ^2_{\mathrm{p}}$	${\mathrm{p}}_{\mathrm{indented}}$ (1.0 mm)	95% C.I.	
					Lower limit	Upper limit
Bare finger	${F}_{8,88} = 23.32$	$< 0.001$ ***	0.68	0.69	0.62	0.75
PM1	${F}_{8,88} = 29.29$	$< 0.001$ ***	0.73	0.45	0.34	0.55
PM2	${F}_{3.9,43.3} = 23.77$	$< 0.001$ ***	0.68	0.21	0.11	0.31
PM3	${F}_{3.6,39.2} = 22.13$	$< 0.001$ ***	0.67	0.20	0.08	0.31

All main effects of touch conditions were significant. *** and C.I. denote $p<0.001$ and confidence interval, respectively.

**Table 2:** Statistical analysis reveals the significant correlations between the rank of indentation magnitude of the surface geometry and Shannon entropy value. *, **, *** denote *p* < 0.05, 0.01, 0.001, respectively.

Perception	Touch condition	Number of action potentials				Entropy (4 ms)			
		$\uptau$	*p* value	$\uprho$	*p* value	$\uptau$	*p* value	$\uprho$	*p* value
Indentation	PM1	0.67	0.01 *	0.78	0.01 *	0.78	0.004 **	0.88	0.002 **
	PM2	0.33	0.21	0.38	0.31	0.78	0.004 **	0.92	0.001 ***
	PM3	0.33	0.21	0.43	0.24	0.83	0.002 ***	0.95	$< 0.001$ ***

## Materials and methods

### Psychological experiment

#### Participants

Twelve participants (age range 19–22 years, six females; all right handed) joined the psychological experiment. The experimental protocol was approved by an ethical committee for human experiments at Shonan Fujisawa Campus of Keio University (approval number 192). Each participant gave informed consent based on the Declaration of Helsinki (1964) prior to participation. All methods were performed in accordance with the relevant guidelines and regulations.

#### Tactile stimulus

We used a fishbone-like pattern^14^ of various intervals between adjacent ribs (Fig. 1a). The fishbone surface pattern was described by three parameters: (1) the width of the central strip, (2) width of a rib, and (3) the distance between adjacent ribs. The central strip width was 4 mm, which is within the range of optimal widths for perceiving the contrast in stimulus intensity between the central and adjacent areas based on a previous study^14^. The rib thickness was 1.0 mm, and the rib intervals were 0.2, 0.4, 1.0, 1.4, 1.8, 2.0, 2.2, 3.0, or 4.0 mm. Each surface pattern was generated using a raised spot ultraviolet printing technique, and the resulting height of the black area (Fig. 1a) was confirmed to be approximately 0.01–0.02 mm on average.

We used a passive pin matrix (Fig. 1b; see Nakatani et al.^40^) to present vibrotactile spatial patterns, as well as rubbing the surface directly with a bare finger. This was done in an apparatus to extract height information from the contact surface beneath the pin matrix. The supporting plate contained holes 0.9 or 1.9 mm in diameter so that each pin could move up and down smoothly, according to the contact surface height beneath the pin matrix. The advantage of using the pin matrix is that it restricts the amount of horizontal displacement of the finger skin. PM1 had 21 $\times$ 21 pins (441 pins in total) with a center-to-center spacing of 1.0 mm; PM2 and PM3 had 11 $\times$ 11 pins (121 pins in total) with a center-to-center spacing of 2.0 mm.

#### Procedures

Each participant entered the room 10 min before the experiment so that the participant’s hands would be at room temperature during the experiment. Participants sat in a comfortable chair and were asked to wear an eye mask during the experiment. The experimenter instructed the participant to touch various tactile stimuli with either a bare finger or the specified pin matrix that was provided by the experimenter. During the experiment, participants conducted a pairwise comparison task. In a trial, participants examined two different tactile stimuli in a trial and were asked to state which one they felt had the deeper indentation (concave shape) in its center in a two-alternative forced-choice fashion. Participants were asked to keep their finger strokes in tempo with a metronome (75 beats per minute) and for a distance of approximately 40 mm between the two ticks of the metronome, such that the velocity of the fingertip was controlled at approximately 50 mm/s. Under the pin matrix condition, the participants moved the pin matrix over the tactile stimulus. Participants were asked to hold the side of the pin matrix with their thumb and middle finger to move the pin matrix and to place their index finger lightly in the center of the pin matrix surface to feel the tactile stimulus. A wooden ruler was used to maintain a parallel orientation of the finger and pin matrix to the long axis of the central strip. Since the diameter of holes in a pin-matrix holder was larger than the pin diameter, the pins moved up and down smoothly. We did not observe any striking, catching, or sticking behavior of pins.

Thirty-six possible tactile stimulus pairs were examined in one session, and two sessions were examined in a block. In a block, the experimenter asked each participant to touch the presented tactile stimuli under one of four touch conditions (bare finger, with PM1, PM2, or PM3). Within a block, participants used the same touch condition. In other words, participants did not compare tactile stimuli under different touch conditions. The order of the presented tactile stimulus pair was randomized between blocks for each participant. The order of touch conditions was also randomized between participants. The participants rested for 5 min every 30 min to avoid sensory fatigue. The experiment lasted 2 h on a given day and was completed over 4 days.

#### Data analysis

We conducted six trials for each touch condition, but we excluded the first two trials of data because the participants took time to get used to the experimental protocol. The mean probability of answering more indentation was used in one-way repeated analysis of variance (ANOVA) to observe the within-participant factor (rib interval). When the sphericity was violated, we used Greenhouse-Geisser correction to calculate the number of degrees of freedom of the F distributions. We also calculated the relative magnitude of indentation in the fishbone pattern by employing Thurstone’s model^41^. Kendall and Spearman rank correlation coefficients were calculated between the psychological data (the rank of mean probability of answering more indented) and numerical results (the rank of temporal frequency or Shannon entropy of sensory neuron responses from receptive fields). Statistical analysis was conducted using IBM SPSS Statistics (Version 25).

### Mathematical model

#### Spatial sampling using pin matrix

In the mathematical model, the fishbone pattern used in the experiment (Fig. 1a) is represented as a two-dimensional (2D) region in which the backbone lies along the *y* axis and the ribs along the *x* axis. The backbone has a width of $L_{\mathrm {w}}$, and the ribs are repeated continuously in the *y* direction with rib thickness $L_1$ and rib-rib spacing $L_2$, which makes the spatial rib period $L_1+L_2$. Then, the height of the fishbone pattern at (*x*, *y*), denoted by $\Phi (x,y)$, is given by

1 $$\begin{aligned} \Phi (x,y) = {\left\{ \begin{array}{ll} h_0, &{} -\dfrac{L_{\mathrm {w}}}{2}< x< \dfrac{L_{\mathrm {w}}}{2} \quad \text {or} \quad k (L_1+L_2)\le y < L_1 + k (L_1+L_2), \\ 0, &{} \text {otherwise}, \end{array}\right. } \end{aligned}$$

where $h_0$ is the height of the fishbone pattern and *k* takes all integer values.

Each pin in the pin matrix moves in the *z* direction when scanning along the fishbone pattern in the *y* direction. Here, we choose a coordinate that moves with the pin matrix: we consider the stationary pin matrix on the moving fishbone pattern $\Phi (x, y-Vt)$, where *V* is the scanning speed. Given a 2D pin coordinate $(x_p, y_p)$, the vertical displacement $h(x_p, y_p)$ of the pin is calculated by taking into account the spherical shape of the pin tip, as follows. When $\Phi (x_p, y_p)=h_0$, the displacement coincides with the height of the fishbone pattern: $h(x_p, y_p)=h_0$. When $\Phi (x_p, y_p)=0$, the displacement depends on the distance $\xi$ between the pin tip $(x_p, y_p)$ and the nearest point of the fishbone pattern, denoted by $(x^*, y^*)$. In the present fishbone pattern, there are only four possibilities: $(x^*, y^*)=(x_p\pm \xi , y_p), (x_p, y_p\pm \xi )$. Then, the displacement is determined by

2 $$\begin{aligned} h(x_p, y_p) = {\left\{ \begin{array}{ll} h_0 + \sqrt{r_p^2 - \xi ^2} - r_p, &{} \xi < \xi ^*, \\ 0, &{} \text {otherwise}, \end{array}\right. } \end{aligned}$$

where $r_p=d_2/2$ is the radius of curvature of the pin tip and $\xi ^*=\sqrt{2r_ph_0-h_0^2}$ is the distance within which the pin is in contact with an edge of the printed pattern. Note that we assume $r_p \ge h_0$; if $r_p < h_0$, then the pin cannot overcome the bump. Thus, the pin displacement *h*(*x*, *y*) depends on not only the fishbone pattern $\Phi (x,y)$ but also the pin radius $r_p$.

#### Mechanical displacement of the skin

The pin displacement is given to the fingertip and converted to mechanoreceptor deformation. Since the mechanoreceptor lies beneath the epidermis and close to the surface ($\sim 0.4$ mm), we simplify the detailed elastic effects as follows: The fingertip that is in contact with a pin with displacement *h*(*t*) at time *t* is assumed to have the same amount of vertical displacement *h*(*t*), which then causes the mechanoreceptor to deform. We assume a linear relationship between the fingertip displacement *h*(*t*) and the mechanoreceptor channel displacement $\sigma (t)$, namely, $\sigma (t)=\chi h(t)$, where $\chi$ is the conversion ratio of the input stimulus and the channel displacement, chosen appropriately as a free model parameter.

#### Response of mechanosensitive channels in a mechanoreceptor

Mechanical stimulation of mechanoreceptors can produce receptor currents that pass through the cell membrane of mechanoreceptors. Receptor currents are achieved by the function of mechanosensitive channels located at the membrane of mechanoreceptors^42^. As a mechanosensitive channel, Piezo 2 is widely recognized in mammalian mechanoreception^42^ and has been observed in representative mechanoreceptors^43–45^. The Piezo 2 channel has a rapidly adapting mechanosensitive current, whose time constant is less than 10 ms^42,44^. Inactivation and adaptation properties in mechanosensitive currents have been pointed out as being two of the main properties of receptor currents^46^: Inactivation means that the receptor currents diminish when the two subsequent stimuli are given, while adaptation means that the stimulus threshold, above which a receptor current is triggered, becomes greater when a continuous stimulus is applied.

Prešern et al. proposed a mathematical model of a mechanoreceptor’s conductance that reflects these two properties^24^: They considered the mechanoreceptor conductance *g*(*t*) in terms of the channel open probability *p*(*t*) and the channel inactivation probability *q*(*t*), namely,

3 $$\begin{aligned} g(t) = g_{\mathrm {max}}p(t)\big (1-q(t)\big ), \end{aligned}$$

where $g_{\mathrm {max}}$ is the maximal conductance of the receptor current. The probabilities *p*(*t*) and *q*(*t*) are governed by

4 $$\begin{aligned} \tau _p\frac{\mathrm {d}p}{\mathrm {d}t}&= - p + \frac{1}{1+\exp [-k_p(\sigma (t) -x_p-\alpha _p q)]}, \end{aligned}$$

5 $$\begin{aligned} \tau _q\frac{\mathrm {d}q}{\mathrm {d}t}&= - q + \frac{1}{1+\exp [-k_q(\sigma (t) -x_q)]}, \end{aligned}$$

where *x* is the strength of the mechanical stimulus and affects both the channel open and inactivation probabilities, while $\tau _p$, $\tau _q$, $x_p$, $x_q$, $k_p$, $k_q$, and $\alpha _p$ are constants. The stimulus applied to the mechanoreceptor is represented by $\sigma (t)$, which in this model is given by the channel displacement $\sigma (t)$.

In summary, a stimulus from fingertip deformation is converted into an input $\sigma (t)=\chi h(t)$ that induces an electrical response from a mechanoreceptor as the conductance *g*(*t*). Supplementary Fig. S2 shows the typical behavior of the channel state probabilities *p*(*t*) and *q*(*t*) and the resulting mechanoreceptor conductance *g*(*t*) when a rectangular input signal of *h*(*t*) is provided.

#### Integration of mechanoreceptor response into sensory neuron responses

We consider a neural response only at a spike initiation zone^47^. We also assume that the receptor potentials (or generator potential in^29,47,48^) from branched sensory neurons can be integrated at a spike initiation site, where signals from the receptors are integrated into the input current that, when sufficiently strong, initiates a nerve pulse as an output signal in the sensory neurons. We do not consider the spatial degree of freedom needed to describe the traveling of pulses. We describe neural activity with the Hodgkin–Huxley model^25,49^ to simulate an action potential in a sensory neuron. In this model, neural dynamics are described by four variables: the action potential of the membrane (*v*), the activation variables of the potassium channel (*m*) and sodium channel (*n*), and the inactivation variable of the sodium channel (*h*). Their dynamics are governed by

6 $$\begin{aligned} C_{\mathrm {m}}\frac{\mathrm {d}v}{\mathrm {d}t}&= - \overline{g}_{\mathrm {K}}n^4 (v - v_{\mathrm {K}}) - \overline{g}_{\mathrm {Na}}m^3 h (v - v_{\mathrm {Na}}) - \overline{g}_{\mathrm {L}}(v - v_{\mathrm {L}}) - I_{\mathrm {stim}}, \end{aligned}$$

7 $$\begin{aligned} \frac{\mathrm {d}m}{\mathrm {d}t}&= \alpha _m(v)(1-m) - \beta _m(v)m, \end{aligned}$$

8 $$\begin{aligned} \frac{\mathrm {d}n}{\mathrm {d}t}&= \alpha _n(v)(1-n) - \beta _n(v)n, \end{aligned}$$

9 $$\begin{aligned} \frac{\mathrm {d}h}{\mathrm {d}t}&= \alpha _h(v)(1-h) - \beta _h(v)h, \end{aligned}$$

where $C_{\mathrm {m}}$ is the membrane capacitance, $v_{\mathrm {K}}$, $v_{\mathrm {Na}}$ and $v_{\mathrm {L}}$ are the equilibrium potentials of potassium, sodium, and leak channels, respectively, and $\overline{g}_{\mathrm {K}}$, $\overline{g}_{\mathrm {Na}}$ and $\overline{g}_{\mathrm {L}}$ are the corresponding conductances of these channels. The stimulus current $I_{\mathrm {stim}}$ represents the input from the receptors connected to the neuron. Here, we consider the simplified situation in which the mechanoreceptor channels in individual receptors are connected directly to the neuron, and we model the stimulus current $I_{\mathrm {stim}}$ as the sum of the receptor currents $I_j$ from $N_{\mathrm {r}}$ receptors;

10 $$\begin{aligned} I_{\mathrm {stim}}&= \sum _{j=1}^{N_{\mathrm {r}}}I_j =\sum _{j=1}^{N_{\mathrm {r}}} g_j(t)(v - v_{\mathrm {eq}}+ v_{\mathrm {offset}}), \end{aligned}$$

where $g_j(t)$ is the conductance of receptor *j* calculated using (3) and $v_{\mathrm {eq}}$ is the equilibrium potential of the membrane. The offset potential $v_{\mathrm {offset}}$ is introduced to ensure that the change in the ion conductance from zero is sufficient to initiate an action potential when $v=v_{\mathrm {eq}}$. The functions $\alpha _m$, $\alpha _n$, $\alpha _h$, $\beta _m$, $\beta _n$ and $\beta _h$ are given as follows^25^:

11 $$\begin{aligned} \begin{aligned} \alpha _m(v)&= A_1 \frac{B_1-v}{\exp \left( \frac{B_1-v}{C_1}\right) - 1},&\beta _m(v)&= A_4 \exp \left( - \frac{v}{C_4} \right) , \\ \alpha _n(v)&= A_2 \frac{B_2-v}{\exp \left( \frac{B_2-v}{C_2} \right) -1},&\beta _n(v)&= A_5 \exp \left( - \frac{v}{C_5} \right) , \\ \alpha _h(v)&= A_3 \exp \left( - \frac{v}{C_3} \right) ,&\beta _h(v)&= \frac{A_6}{\exp \left( \frac{B_6-v}{C_6} \right) + 1}, \end{aligned} \end{aligned}$$

where $A_1=0.1$ ms$^{-1}$, $B_1=25$ mV, $C_1=10$ mV, $A_2=0.01$ ms$^{-1}$, $B_2=10$ mV, $C_2=10$ mV, $A_3=0.07$ ms$^{-1}$, $C_3=20$ mV, $A_4=4$ ms$^{-1}$, $C_4=18$ mV, $A_5=0.125$ ms$^{-1}$, $C_5=80$ mV, $A_6=1.0$ ms$^{-1}$, $B_6=30$ mV, and $C_6=10$ mV. We solve these equations numerically by using the fourth-order Runge–Kutta method with the following parameter values: $g_{\mathrm {max}}= 1.0\ \mathrm{m}\Omega ^{-1}$; $\tau _p= 2.5$ ms; $\tau _q= 8.0$ ms; $k_p = 2.6\ \upmu \mathrm{m}^{-1}$; $k_q = 1.2\ \upmu \mathrm{m}^{-1}$; $x_p = 4.0\ \upmu \mathrm{m}$; $x_q = 6.0\ \upmu \mathrm{m}$; $\alpha _p = 4.6\ \upmu \mathrm{m}$; $\overline{g}_{\mathrm {Na}}= 120$ mS/cm$^2$; $\overline{g}_{\mathrm {K}}= 36$ mS/cm$^2$; $\overline{g}_{\mathrm {L}}= 0.3$ mS/cm$^2$; $v_{\mathrm {K}}= -12.0$ mV; $v_{\mathrm {Na}}= 115.0$ mV; $v_{\mathrm {L}}= 10.6$ mV; $v_{\mathrm {eq}}= 60.0$ mV; $v_{\mathrm {offset}}= -10.0$ mV; $C_{\mathrm {m}}= 1.0\ \upmu$F/cm$^2$; $\chi = 0.07$. These values were chosen according to the references^24,49^, except for $x_q$ and $\chi$, which were determined through extensive parameter searches (details are shown in Supplementary Fig. S6).

#### Overview of ensembles of sensory neuron responses

The fingertip is considered a 10 mm $\times$ 20 mm rectangular region, on which $N_n=72$ neurons are placed in a square lattice with distance $L_x=L_y=10/6$ mm. Each neuron has a circular receptor field with a radius $r=1$ mm, in which $N_r=4$ receptors are randomly distributed. See Supplementary Fig. S2 . We also confirmed, by performing simulations with five different random distributions of the receptive fields, that the regularity of the distribution of the receptive fields does not significantly affect the firing patterns.

#### Numerical experiment

We performed numerical experiments corresponding to PM1, PM2, and PM3. For each experimental set, we prepared twenty finger samples. Each finger sample contained a different random receptor distribution. The pin matrix with spacing of 1.0 mm (PM1) or 2.0 mm (PM2 and PM3) is represented as a 2D periodic array of pins that covers the entire fingertip. A receptor receives input signals from the vertical movement of a pin when the distance between the receptor and the center of the pin is within $2r_1$ ($r_1=d_1/2$ is the radius of the pin head). The parameters of the fishbone pattern used in each numerical experiment corresponded to those used in the psychological experiment: $h_0=0.1$ mm; $L_{\mathrm {w}}=4.0$ mm; $L_1=1.0$ mm; and $L_2$ values varied from 0.2 to 4.0 mm. The scanning speed *V* was set to 50 mm/s, corresponding to the psychological experimental conditions.

#### Data analysis

Each numerical experiment yields output data as a set of time series produced by all the neural activities represented by the neural output *v*(*t*) from $t=0$ to $t=T=800$ ms. In each neural activity, a firing event is defined as a moment when *v*(*t*) passes a threshold value $v^*$ from below. We set $v^*=40.0$ mV.

We divide the whole simulation time *T* into *n* time intervals. In each interval $\Delta t=T/n$, we count the number of firing events for each neuron. Let $F_i$ be the sum of the firing events from all neurons in $[(i-1)\Delta t, i\Delta t]$ ($i=1, \dots , n$) and $p_i={F}_{i}/\sum _{i=1}^n F_i$, the fraction of firing events. Then, the Shannon entropy *H*, which measures the degree of randomness, is defined as

12 $$\begin{aligned} H = -\sum _{i=1}^n p_i \log p_i, \end{aligned}$$

Note that *H* depends on $\Delta t$, which we choose as $\Delta t=4$ ms, and the mean firing rate $\bar{F}$ per neuron, averaged over $N_n$ neurons, is given by

13 $$\begin{aligned} \bar{F} = \frac{1}{T}\frac{1}{N_n}\sum _{i=1}^n F_i. \end{aligned}$$

## Supplementary Information

Supplementary Information 1.

Supplementary Information 2.

Supplementary Information 3.

Supplementary Information 4.

Supplementary Information 5.

Supplementary Information 6.

Supplementary Information 7.

Supplementary Information 8.

Supplementary Information 9.

Supplementary Information 10.
