# Supplementary figures and images for: Temporal coherency of mechanical stimuli modulates tactile form perception
Source: Sci Rep. 2021 Jun 3;11:11737. doi: 10.1038/s41598-021-90661-1 (PMC8175693; doi:10.1038/s41598-021-90661-1)

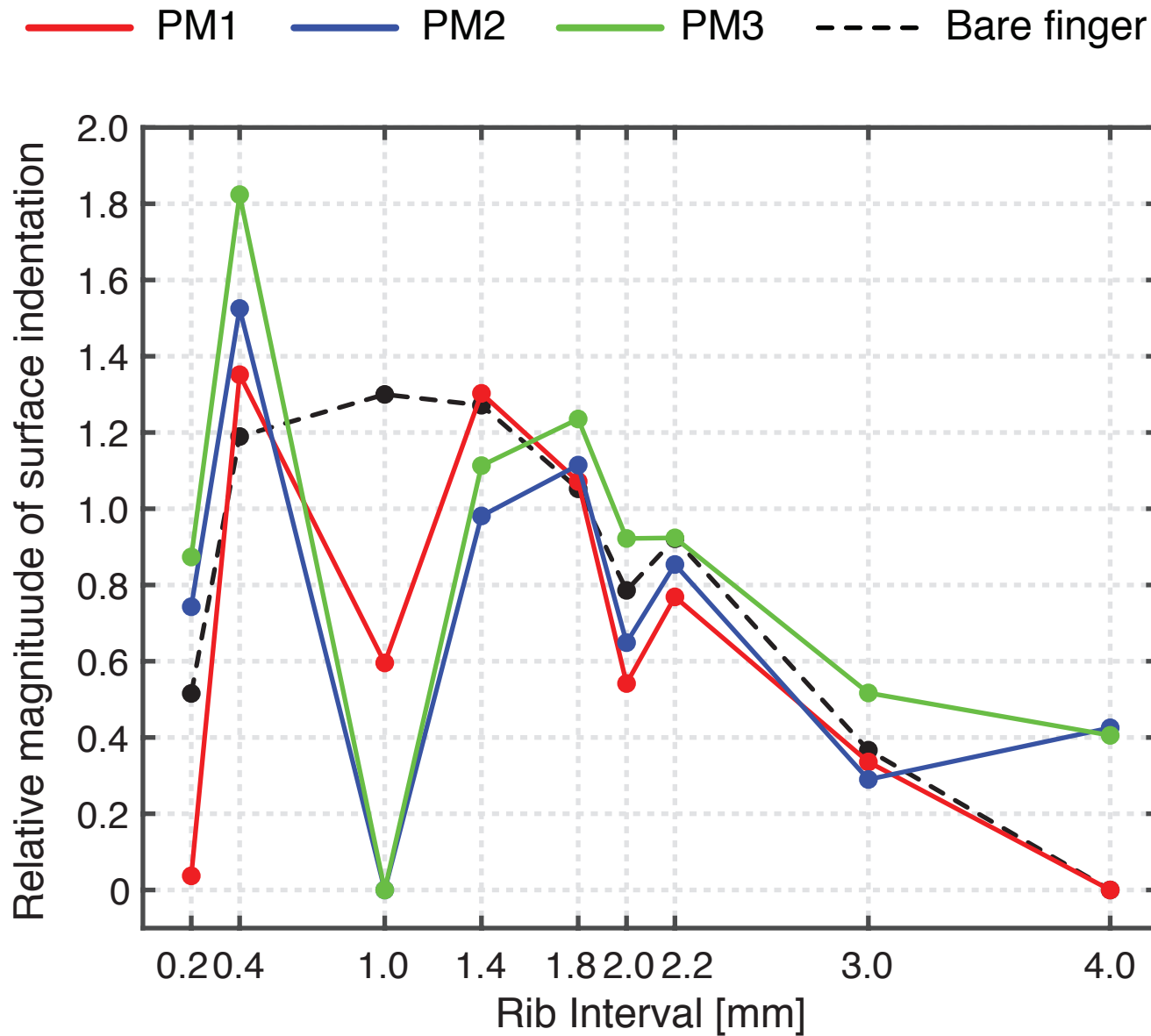

Supplement: Supplementary file 1 — Supplementary Information 1. [file 41598_2021_90661_MOESM1_ESM.pdf]

mechanoreceptors

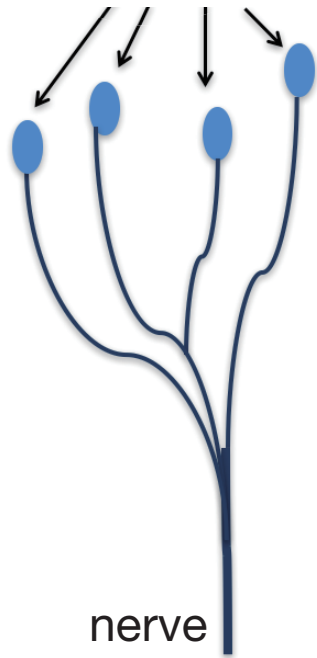

nerve

nerve

mechanoreceptor

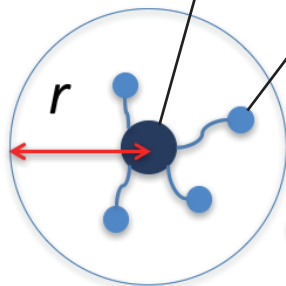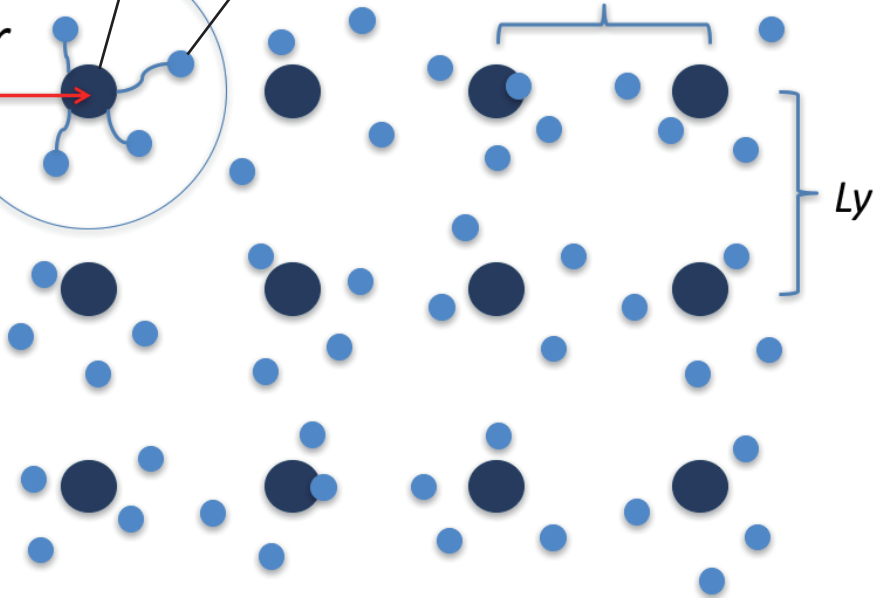

Supplement: Supplementary file 2 — Supplementary Information 2. [file 41598_2021_90661_MOESM2_ESM.pdf]

**a**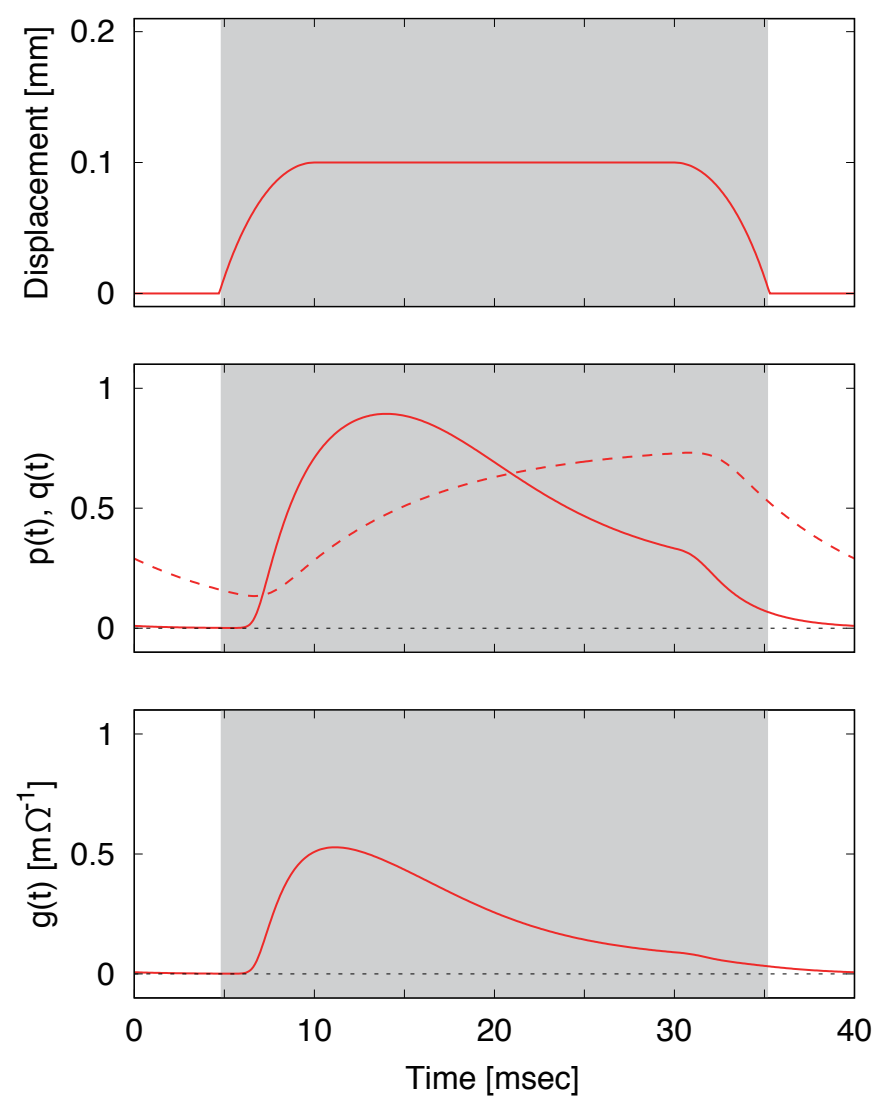**b**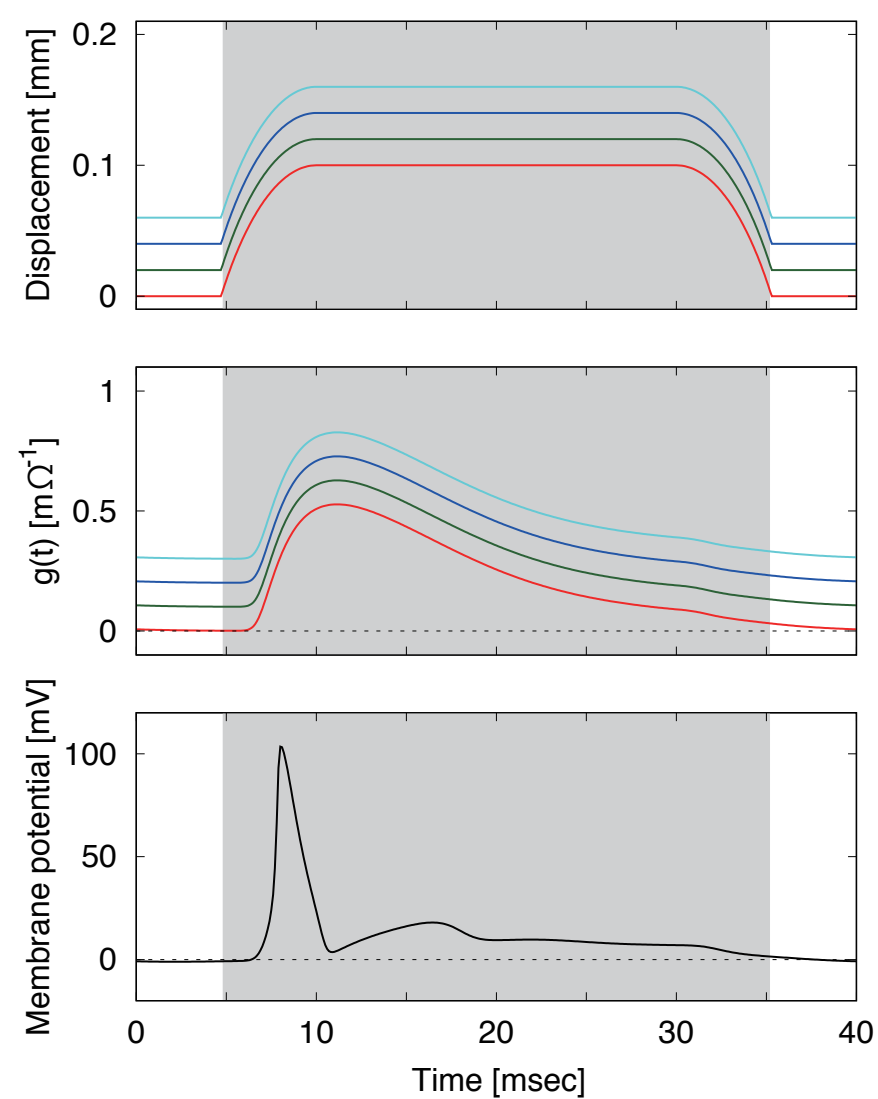

Supplement: Supplementary file 3 — Supplementary Information 3. [file 41598_2021_90661_MOESM3_ESM.pdf]

**a**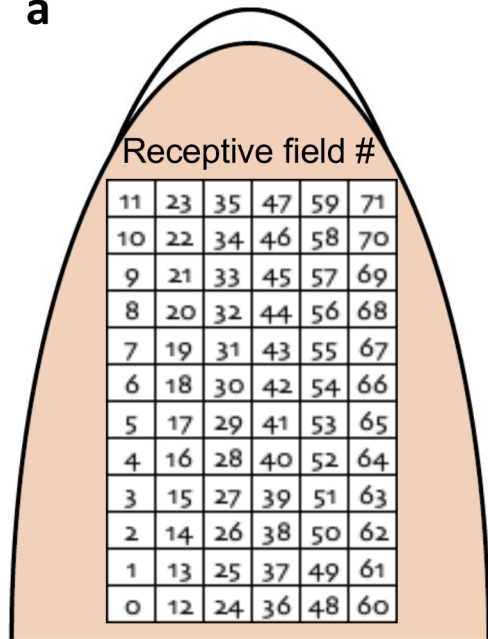**b**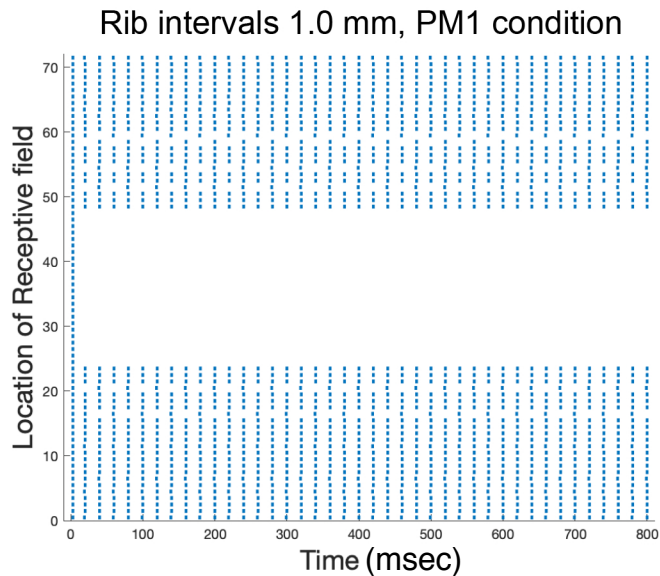**c**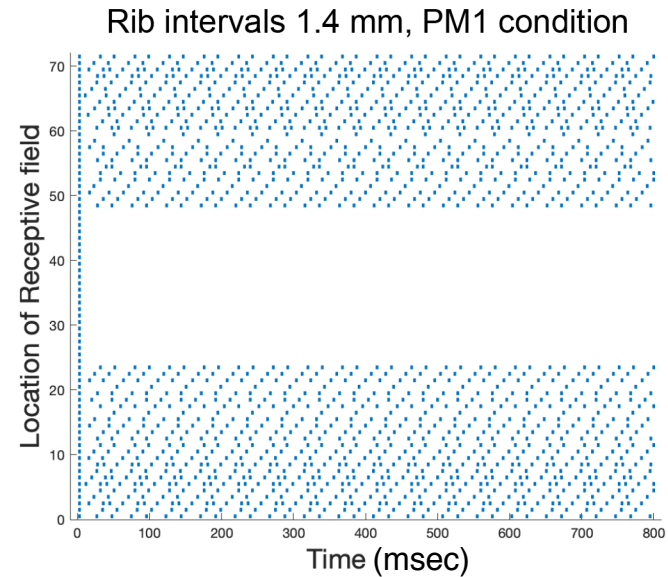

Supplement: Supplementary file 4 — Supplementary Information 4. [file 41598_2021_90661_MOESM4_ESM.pdf]

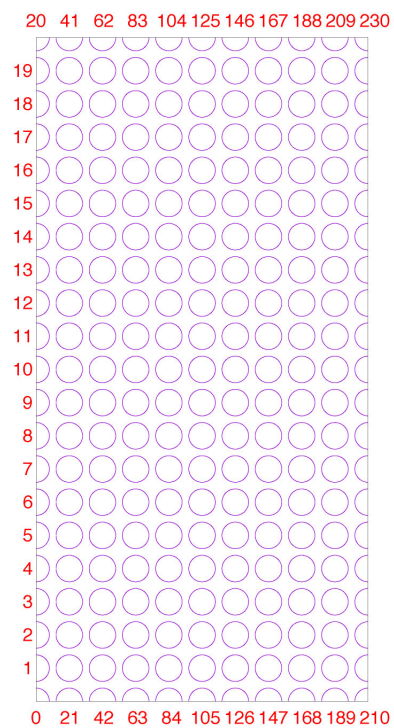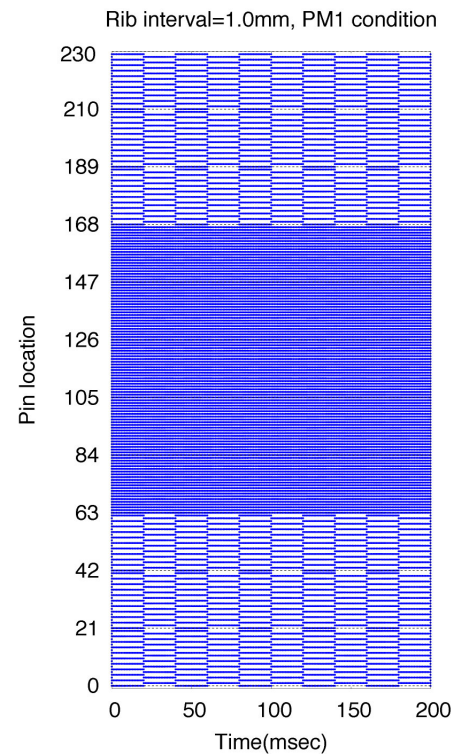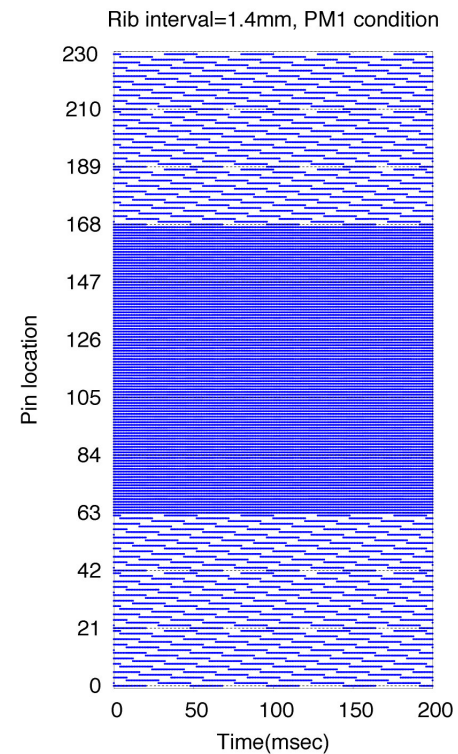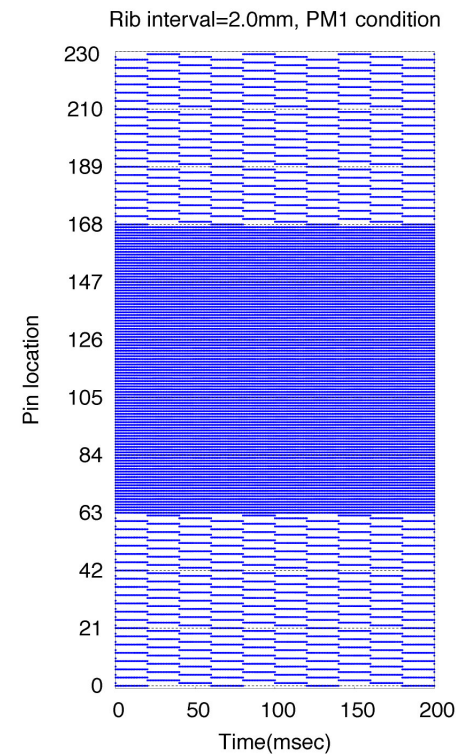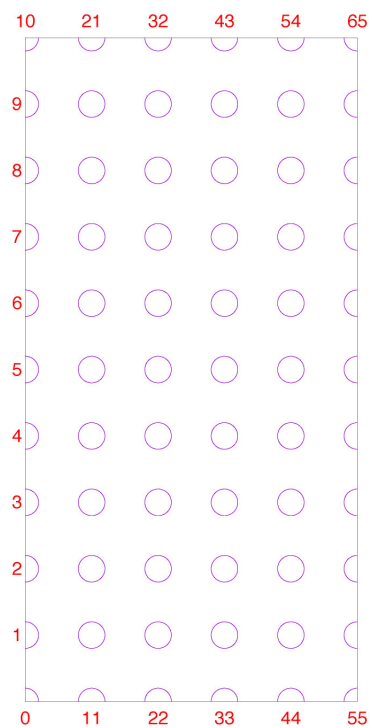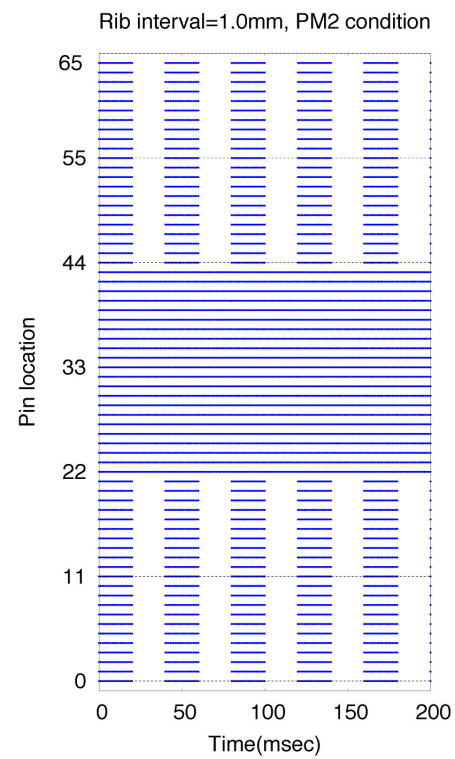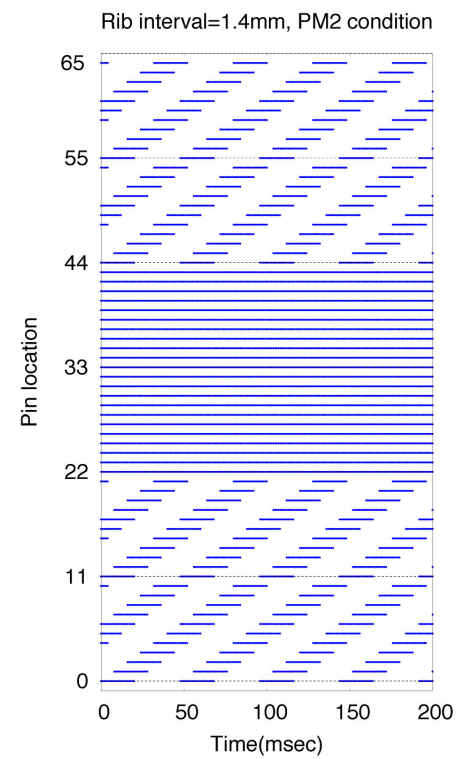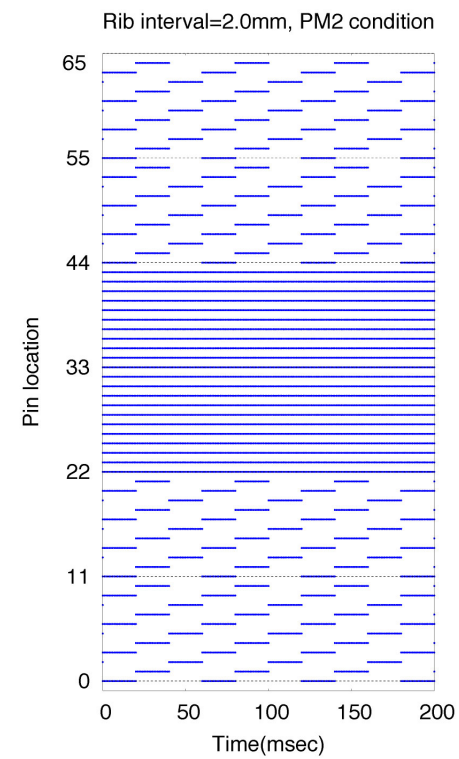

Supplement: Supplementary file 5 — Supplementary Information 5. [file 41598_2021_90661_MOESM5_ESM.pdf]

**a**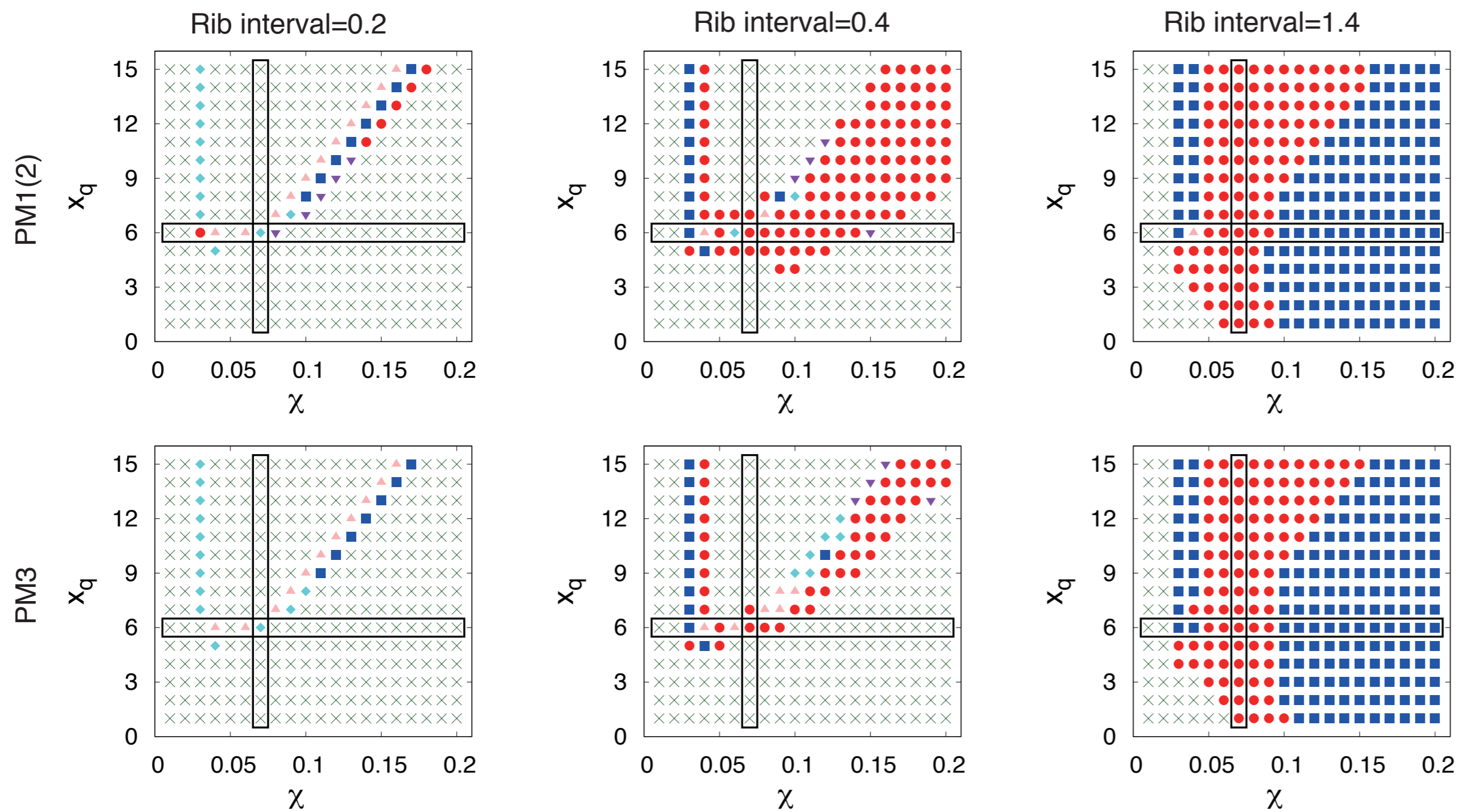**b**

example for profile of membrane potential

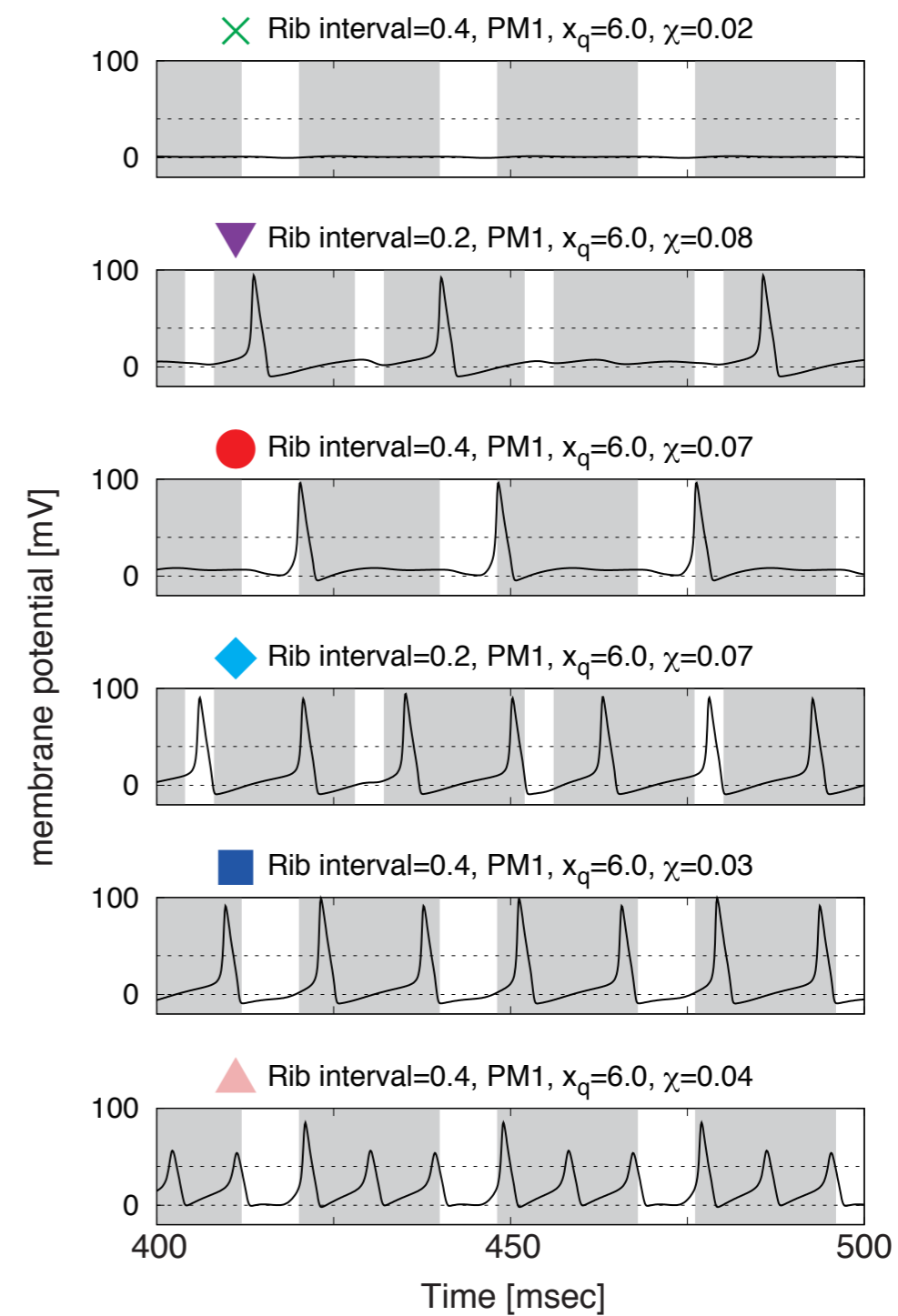

Supplement: Supplementary file 6 — Supplementary Information 6. [file 41598_2021_90661_MOESM6_ESM.pdf]
