# Supplementary material for: Temporal coherency of mechanical stimuli modulates tactile form perception
Source: Sci Rep. 2021 Jun 3;11:11737. doi: 10.1038/s41598-021-90661-1 (PMC8175693; doi:10.1038/s41598-021-90661-1)
Supplement: Supplementary file 7 — Supplementary Information 7. [file 41598_2021_90661_MOESM7_ESM.pdf]

Supplementary Information

Paper Title: Temporal coherency of mechanical stimuli modulates tactile form perception

Authors: Masashi Nakatani, Yasuaki Kobayashi, Kota Ohno, Masaaki Uesaka, Sayako Mogami, Zixia Zhao, Takamichi Sushida, Hiroyuki Kitahata, and Masaharu Nagayama

Supplementary Figure 1 (SupplementaryFigure1.pdf)

Psychological data were plotted in terms of the relative magnitude of the perceived surface indentation according to rib intervals. Thurstone's paired comparison procedure was utilized. All responses from twelve participants (forty-eight answers from twelve participants) were summed to calculate the values. We set the smallest magnitude of the perceived surface indentation as zero in each condition.

Supplementary Figure 2 (SupplementaryFigure1.pdf)

Schematic of mechanoreceptors connected to a sensory nerve in the computational model. Mechanoreceptors were randomly distributed in a plane, and a receptive field consisted of four mechanoreceptors that were closest to the center of the receptive field (left). We assumed that the length of separation between the center of the receptive fields was Lx = Ly = 1.0 mm in the numerical simulation.

Supplementary Figure 3 (SupplementaryFigure3.pdf)

An example of the temporal response of a mechanoreceptor. a. A mechanoreceptor was displaced by a pin (gray area), and two parameters of the mechanosenstive ion channel (p(t) for activation [solid line] and q(t) for inactivation [dashed line]) were changed, resulting in an increase in channel conductance g(t). b. Four mechanorecepotors are connected at a spike initiation site (See Fig. 3d) where the sum of ion currents is integrated. If the sum of the ion currents exceeded the threshold, the spike initiation site could produce an action potential ((b) bottom). Displacements and conductances of four mechanoreceptors (red, green, blue cyan) are plotted with slight shifting for clarity.

Supplementary Figure 4 (SupplementaryFigure4.pdf)

Examples of the response from sensory neurons in computational simulations. (a) Configurations of the receptive field and representative results for (b) 1.0 mm and (c) 1.4 mm rib intervals.

Supplementary Figure 5 (SupplementaryFigure5.pdf)

Configurations of the pin matrices (PM1 [top row] and PM2 [bottom row]) in the left column. Purple circles indicate the area of contacting pins with pin numbers (indicated in red). From the second to the fourth columns, the displacement trace of each pin is shown for three different rib intervals (1.0, 1.4, and 2.0 mm). Blue indicates the temporal trace of the maximum displacements.

Supplementary Figure 6 (SupplementaryFigure6.pdf)

An overview of the phase diagram for determining free parameters χ and x_q that govern the characteristics of neural responses. (a). For three different rib intervals and two different pin conditions, numerical simulations were performed by varying χ and x_q For each parameter set, we simulated the response of a single neuron with four receptors, which were on the same pin and received identical signals. The time profiles of the membrane potentials were calculated and classified into six distinct patterns according to the mean number of responses during one period of input signals, denoted by p: $p=0$ (cross), $0<p<1$ (inverted triangle), $p=1$ (circle), $1<p<2$ (diamond), $p=2$ (square), and $p>2$ (triangle). Typical profiles are shown in (b). We chose the parameter set from the parameter region in which (i) membrane potentials that were in accordance with the pin displacement (circle) were observed in as many cases as possible, and (ii) membrane potentials were preserved even in the case of rib interval = 0.2. Based on this observation, we set the free parameters as $\chi=0.07$ and $x_q=6.0\ \mu m$.

Supplementary Videos 1-3

SupplementaryVideo1.mp4

SupplementaryVideo2.mp4

SupplementaryVideo3.mp4

These videos demonstrate the time lapse of how seventy-two receptive fields (large circles) responded to surface asperity of the fishbone pattern. Four mechanorecepotrs (small circle) were connected to each receptive field. These video files are numerical simulations for scanning the fishbone pattern of 1.0 mm (Supplementary Video 1), 0.4 mm (Supplementary Video 2), under PM1 (1.0 mm spacing) condition and the fishbone pattern of 1.0 mm (Supplementary Video 3) under PM2 (1.0 mm spacing) condition. Note that receptive fields in adjacent areas responded simultaneously for scanning the fishbone pattern of 1.0 mm for PM 1 (Supplementary Video 1) and for PM2 (Supplementary Video 3). Bottom plot indicates the time-lapse of the pin displacement in the mechanoreceptor at the most left bottom.
